# Attitudes of Veterinary Teaching Staff and Exposure of Veterinary Students to Early-Age Desexing, with Review of Current Early-Age Desexing Literature

**DOI:** 10.3390/ani8010003

**Published:** 2017-12-25

**Authors:** Alannah Jupe, Jacquie Rand, John Morton, Sophie Fleming

**Affiliations:** 1 School of Veterinary Science, The University of Queensland, Gatton, QLD 4343, Australia; j.rand@uq.edu.au (J.R.); john.morton@optusnet.com.au (J.M.); sophiefleming@gmail.com (S.F.); 2 Australian Pet Welfare Foundation, Kenmore, QLD 4069, Australia; jacquie@petwelfare.org.au; 3 Jemora Pty Ltd., Geelong, VIC 3220, Australia

**Keywords:** pet population control, teaching staff, veterinary students, desexing, early age desexing, sterilization, attitudes, spay, neuter, gonadectomy, cat, dog

## Abstract

**Simple Summary:**

A substantial proportion of cats and a smaller proportion of dogs entering animal shelters are juveniles. Cats are prolific breeders and can be pregnant by 4 months of age, although the traditional desexing age in veterinary practice is 6 months for dogs and cats. Understanding what veterinary students across Australia and New Zealand are being taught in regards to early age desexing (EAD) is necessary to determine the reasons why EAD is not utilized in client-owned cats and dogs more frequently. There are no current studies documenting the exposure of new veterinary graduates to EAD, nor the opinions to EAD of the academics teaching these students. Of staff teaching veterinary students in Australia and New Zealand in 2015, a majority (64%) did not advocate EAD in their teaching of students. Only three out of eight universities provided a majority of students with exposure to EAD procedures before graduation, and only two of these allowed most students to perform EAD.

**Abstract:**

Approximately 50% of cats admitted to Australian shelters are kittens, and 26% of dogs are puppies, and, particularly for cats, euthanasia rates are often high. Cats can be pregnant by 4 months of age, yet the traditional desexing age is 5–6 months, and studies in Australasia and Nth America reveal that only a minority of veterinarians routinely perform early age desexing (EAD) of cats or dogs, suggesting they are not graduating with these skills. This study aimed to describe the attitudes of veterinary teaching staff in Australian and New Zealand universities towards EAD, and to determine if these changed from 2008 to 2015. It also aimed to identify students’ practical exposure to EAD. Most (64%) of the 25 participants in 2015 did not advocate EAD in their teaching and, in their personal opinion, only 32% advocated it for cats. Concerns related to EAD cited by staff included anesthetic risk, orthopedic problems, hypoglycemia, and, in female dogs, urinary incontinence. Those who advocated EAD cited benefits of population control, ease of surgery and behavioral benefits. Only three of the eight universities provided a majority of students with an opportunity to gain exposure to EAD procedures before graduation, and in two of these, most students had an opportunity to perform EAD. In conclusion, most veterinary students in Australia and New Zealand are not graduating with the knowledge or skills to perform EAD, and have little opportunity while at university to gain practical exposure. Welfare agencies could partner with universities to enable students to experience EAD.

## 1. Introduction

Thousands of dogs and cats are admitted to animal shelters and municipal pounds in Australia each year [1,2,3] and, as in other countries, a large number of healthy animals are subsequently euthanized [1,2,3,4,5,6]. For example, in 2013–2014, the year prior to this study, the largest welfare organization in Australia, the Royal Society for Protection of Animals (RSPCA), admitted 49,166 cats and 45,954 dogs and 31% and 15%, respectively, were euthanized [2]. Of the cats entering RSPCA shelters between 2006 and 2010, over half (54%) were kittens [1], and approximately 40% of kittens were from client-owned queens [6], suggesting that intentional or unintentional breeding of owned cats is a significant source of unwanted kittens. In contrast, approximately 26% of dogs entering RSPCA shelters were puppies 6 months of age or less [2].

Over 90% of owned cats in Australia are desexed [7] but, as in North America, 12–20% have a litter before the procedure, contributing to the production of unwanted kittens [8,9,10]. Cats are prolific breeders, and most owners are unaware their cat can reach puberty by 4 months of age (depending on breed), well before the traditional desexing age of 6 months [10,11,12,13,14]. In a Western Australian study, of cats presented for discounted microchipping in 2012 and 2013, only 28% and 49%, respectively, of those less than 2 years old were desexed, compared to 93% and 97% of those aged more than 2 years [7]. This is further evidence that, although most owned cats in Australia are eventually desexed, this is commonly performed after sexual maturity. The age of puberty for dogs can vary between 6–14 months, but unlike cats, the traditional desexing age of 6 months for dogs reduces the risk that pubertal bitches will have unwanted pregnancies, because desexing procedures are generally performed before sexual maturity [13,14,15].

Desexing of cats and dogs is an effective method of population control [16] and early age desexing (EAD)—that is, performing desexing before approximately 24 weeks of age, and/or before the first estrus—is utilized by many animal shelters [17,18]. Routine incorporation of desexing in the initial health program of domestic pets at 12–14 weeks of age, especially for cats, may reduce the number of unplanned pregnancies [19]. EAD is endorsed by many international organizations, including the British Small Animal Veterinary Association, and Australia’s Royal Society for the Prevention of Cruelty to Animals (RSPCA) [20]. However, the American Veterinary Medical Association recently revised its position on EAD, especially for dogs, after several recent reports of increased frequency of orthopedic and neoplastic conditions in dogs desexed at less than 12 months of age [21,22,23,24]. The American Veterinary Medical Association currently recommends cats not intended for breeding should be desexed prior to 5 months of age and that the decision of when to desex an individual dog or cat should be made by the practicing veterinarian [21]. Irrespective of support for EAD, even for cats, it is only performed by a minority of veterinarians in general practice in Australia [25,26], New Zealand [25] and USA [27]. It is unknown what veterinary students are learning and whether skills for EAD are being developed before graduating, and what are the predominant attitudes of veterinary teaching staff towards EAD. Graduating veterinarians should have knowledge of the advantages and disadvantages of EAD, and should graduate with skills to perform it, especially for cats, as research shows that EAD is a safe procedure to perform if the veterinarian (surgeon and anesthetist) possesses the appropriate skills [28], and no significant adverse effects have been reported [5,29]. Better understanding of attitudes of veterinary teaching staff to EAD would provide opportunities for animal welfare agencies to engage with universities to assist in developing competency in EAD for graduating veterinary students.

The aim of this study was to describe the attitudes and beliefs of Australian and New Zealand veterinary teaching staff towards EAD in shelter and client-owned animals, and to determine (i) if EAD is being recommended to students within veterinary schools; (ii) specific reasons why teachers advocate EAD or not when teaching; and (iii) how much exposure students gain to early age desexing prior to graduating. We also aimed to compare these between 2008 and 2015. 

## 2. Materials and Methods

The University of Queensland’s Institutional Human Research Ethics Committee approved the study (No. 2015000380), and in 2008 and 2015, heads/deans of all veterinary schools in Australia and New Zealand were contacted, and asked to identify their veterinary staff who were teaching practical, theoretical or anesthetic procedures for desexing to veterinary students. These staff were then contacted via email and asked to participate in the study.

The questionnaire contained the same multiple choice and open-ended questions regarding the participant’s beliefs and attitudes towards EAD in 2008 and 2015, except for nine additional questions in 2015 (Table S1). In both years, participants were asked identify their preferred age bracket—≤3 months, 4–5 months, or ≥6 months—for desexing of client-owned and shelter cats and dogs. They were also asked if they advocated EAD when teaching students, and the possible benefits and risks of EAD. Specific benefits and risks were offered as options along with scope for participants to record other responses. The new questions asked if students were able to view or perform EAD before they graduated, and whether staff themselves performed these procedures. 

In 2015, 37 potentially eligible teaching staff were identified, and all were contacted, of which five were then identified as not teaching desexing to students, three did not respond and 29 agreed to participate and completed questionnaires. Subsequently, four respondents were excluded because they were no longer teaching or not directly teaching desexing to students, leaving 25 respondents. In 2015, participants were from The University of Queensland (*n* = 5), University of Sydney (*n* = 3), University of Melbourne (*n* = 3), Murdoch University (*n* = 5), University of Adelaide (*n* = 4), Charles Sturt University (*n* = 3), James Cook University (*n* = 1), and Massey University (*n* = 1). In 2008, 19 potentially eligible teaching staff were identified and contacted, and 15 completed the questionnaire. Participants were from The University of Queensland (*n* = 4), University of Sydney (*n* = 3), University of Melbourne (*n* = 4), Massey University (*n* = 4) and Murdoch University (*n* = 1). In 2008, the three new Australian veterinary schools which had not yet graduated students were not included (University of Adelaide, Charles Sturt University, James Cook University). There were four staff members who responded in both 2015 and 2008. Thus, 36 staff respondents provided a total of 40 responses when data from 2008 and 2015 were pooled. Graduation details of teaching staff were collected only in 2015.

### Statistical Analyses

For statistical analyses, responses describing the perceived best ages to desex were categorized as ≤5 or >5 months. Proportions of respondents who selected each and proportions that advocated early age desexing were compared between 2008 and 2015. These data were incompletely paired: that is, some subjects responded in both years and others responded in just one year. Most commonly-used methods for analyzing incompletely paired binary data are asymptotic, and so can be unreliable with small samples sizes such as in the current study. We are not aware of exact methods for analyzing incompletely paired binary data so, as only four of the 36 staff responded in both years, we calculated exact two-tailed mid-p *p*-values to test the null hypotheses that the proportions did not differ between 2008 and 2015 disregarding this clustering. The Compare2 module (Version 3.83) in WinPepi (version 11.63, http://www.brixtonhealth.com/) was used [30]. Confidence intervals for differences in proportions between 2008 and 2015 were calculated using a procedure for incompletely-paired binary data [31]. R code was provided by one of the authors of that publication. Confidence intervals referred to as ‘exact unconditional two-sided 95% confidence intervals’ were calculated. These can exhibit stochastic variation and so results for the same data can vary somewhat. Accordingly, both the *p*-values and confidence intervals’ should be considered as approximations. Only descriptive demographic information of respondents is reported because there was a large range of universities of graduation, and relatively few new graduates teaching desexing for meaningful analysis of effect of age on responses. 

## 3. Results

### 3.1. Participants

In this study of Australian and New Zealand veterinary school staff directly teaching anesthetic and surgical aspects of desexing to veterinary students, in 2015, of all 28 teaching staff identified from eight veterinary schools as eligible for the study, 25 (89%) participated. For 2008, of all 19 teaching staff identified in four veterinary schools as eligible for the study, 15 (79%) participated. Out of the 25 participants in 2015, six taught anesthesia-related aspects of desexing to students, 12 taught surgical aspects of desexing and seven taught both. Out of the 15 participants in 2008, eight taught aspects of anesthesia related desexing to students, seven taught surgical aspects of desexing and one taught both. The 25 respondents in 2015 had gradated between 8 and 36 years previously (median 25 years). The most common universities they graduated from were Sydney (8), Melbourne (3) and Murdoch (2). Teaching positions varied from intern and tutor to professor, with most in the mid-range of seniority (eight lecturers and 11 senior lecturers). 

### 3.2. Veterinary Teaching Staff Opinions on EAD

In 2015, when all factors were considered, including safety and population control, most staff teaching desexing to veterinary students thought 4–5 months was the best age to routinely perform desexing in male and female client-owned cats, whereas ≤3 months was most commonly thought best for shelter-owned cats (Table 1). In regards to desexing in dogs, most participants selected ≥6 months of age as the best age to desex client-owned animals. In contrast, the most commonly selected best age to desex female shelter dogs was ≥6 months for female dogs and ≤3 months for males. When compared with 2008, lower proportions of veterinary teaching staff in 2015 advocated desexing at ≤5 months for male dogs in shelters, and for female and male cats in shelters (*p* = 0.044 for each). 

In 2015, when asked “in your personal opinion of EAD, do you advocate its use”, a minority of staff members advocated EAD in cats (32%) and dogs (20%), and there was some evidence that both were lower than in 2008 in male and female cats and male dogs (*p* = 0.066 to 0.077; Table 2). The percentage advocating EAD in female dogs remained the same, a minority of 20%. In their teaching of desexing to veterinary students in 2015 and 2008, only 24% and 20%, respectively, advocated the use of EAD for both dogs and cats, regardless of ownership or sex of the animal (Table 3). A minority of staff did not firmly confirm if they advocated EAD or not in their teachings, noting that it depended on the species and situation of the animals (2015: 12%; 2008: 27%).

The reasons veterinary teaching staff advocating EAD gave for doing so were similar in 2015 and 2008. In 2015 the main reasons staff advocated EAD were population control (89% or 8/9), ease of surgery (78% or 7/9) and quicker recovery (44% or 4/9). In 2008, the major reason veterinary teaching staff advocated EAD was population control (71% or 5/7) (Table 4).

The major reasons veterinary teaching staff not advocating EAD in 2015 gave for not doing so were anesthetic risks (63% or 12/19), and the increased risk of one or more medical problems including hip dysplasia in large breed dogs, cranial or anterior cruciate ligament rupture, urinary incontinence in female dogs and hypoglycemia. Reasons were similar in 2008 (Table 5). When staff who answered “no” to advocating EAD were asked, “under what situations would you support EAD”, of the 27 responses for 2008 and 2015 pooled, for 16 (59%), the respondent volunteered that they would advocate EAD in a shelter situation where animals were being reclaimed or rehomed. 

### 3.3. Practical Exposure of Students to EAD

Of the eight Australian and New Zealand veterinary schools, in 2015, only three (37%) provided an opportunity in practical sessions or in final year rotations for the majority of veterinary students to witness EAD procedures before they graduated, and, in two of these, the majority of students were able to perform EAD. In two other universities, it was commented that a minority of students might get exposure to desexing between 4 to 6 months, depending on the age of animals available for desexing teaching sessions. Half (four) of the universities provided no opportunity to witness or perform EAD procedures (Table 6). Three universities had working associations with animal welfare agencies such as the RSPCA or the Lost Dogs Home, or animal rescue or fostering groups, which enabled these universities to access animals for EAD when teaching desexing. 

When asked in 2015 how many puppies or kittens 4 months or younger they had desexed in the last 12 months, with or without students, only 32% (8/25) of respondents had performed these procedures. All 8 staff members had performed these procedures with students; one had also performed these procedures without students. 

In response to the question asked in 2015 “in your opinion, do you believe implementing routine desexing of client-owned kittens before 4 months of age would result in a measurable reduction in the number of unwanted kittens from owned queens that are surrendered to shelters”, 44% (11/25) of respondents indicated no, 36% (9) said yes, and 20% (5) indicated that they were uncertain.

## 4. Discussion

In this study of attitudes and opinions to EAD of veterinary staff teaching in Australian and New Zealand universities, only a minority in 2015 advocated it for cats (32%), and fewer advocated it for dogs (20%), regardless of sex of animal. When all factors were considered, including safety and population control, the ages most thought best for desexing for client-owned animals were 4–5 months for cats and ≥6 months for dogs, irrespective of sex. In contrast, for shelter animals, their preferred age was most commonly ≤3 months for cats, ≥6 months for female dogs, and ≤3 months for male dogs. Compared to 2008, in 2015, a smaller percentage of staff thought ≤5 months was the best age to desex male dogs in shelters, and male and female cats in shelters, but the confidence intervals were wide. Importantly, in their teaching to veterinary students of desexing for both female and male cats and dogs, in 2015 only 24% advocated the use of EAD, which was similar to the 20% advocating it their teaching in 2008. This indicates that although teaching veterinarians support desexing procedures for cats and dogs, in their teaching of veterinary students, most do not advocate EAD, and few thought the best age for desexing client-owned cats was <4 months. 

Our findings are similar to those from a US study, where 90% of 412 New York veterinarians recommended 5 months of age for client-owned animals, and 3 months of age for shelter animals as the earliest age for desexing. Of the 55% of participants who had learnt to perform EAD, only 20% developed these skills as veterinary students [27]. In a 2013 study of 717 randomly selected practicing veterinarians (35% New Zealanders and 28% Australian), most respondents considered 4 to <5.5 months an appropriate age for desexing in cats (95% for females, 94% for males), and only a minority considered <4 months an appropriate age (33% for females, 30% for males) [25]. In a recent survey of 774 Australian veterinarians, respondents most commonly recommended desexing dogs and cats at 6 months [32]. The traditional age recommended in the veterinary literature for desexing procedures in dogs and cats is between 6 and 9 months [10,11,12]. This reflects the age that veterinarians are comfortable performing anesthesia and surgery, and is likely how they were trained as veterinary students [12]. Our study suggests that veterinary students may not be being educated about the benefits of early age desexing by staff directly teaching components of desexing, both anesthesia and surgery, although our study did not investigate what students may be learning in other course subjects—for example, welfare or professionalism components. Our study also demonstrates that as in USA, a majority of veterinary students are not gaining the practical skills needed to perform EAD.

Although most respondents did not advocate EAD to veterinary students, a majority (78% for cats and 65% for dogs) believed the best age to desex shelter animals was ≤5 months. This might have been because some participants believed that EAD is acceptable if used for population control because it will decrease numbers of healthy animals that are euthanized, but it is unacceptable for client-owned animals due to increases in risk of various adverse outcomes including anesthetic complications, and subsequent medical problems. This disparity in belief about the best age to desex shelter and client-owned animals is not logical if based on concern for subsequent adverse effects on the animal’s health, because both groups of animals are subsequently owned by clients, and there is no reason to suspect that animals obtained from shelters are at greater risk of contributing to overpopulation of pets. It may also reflect teaching staff’s confidence in performing EAD, and their lack of training in this as an undergraduate student, as well as their current expertise, because only 32% had performed desexing of puppies or kittens 4 months or younger in the previous 12 months. 

With the continuing overproduction of kittens, alternative methods of population control besides euthanasia should be implemented, and EAD is one of these [19]. Unplanned litters are one of the biggest sources of animals, in particular kittens, that are admitted to shelters and pounds [19,33]. Unwanted kittens from client-owned queens constituted 40% of kittens being surrendered to RSPCA shelters in Australia [6]. If animals can be prevented from having unplanned litters, for example by being desexed before sexual maturity, it is likely that shelter intake of kittens will decrease. Decreasing shelter intake is the most effective way to decrease numbers of animals euthanized [9]. As veterinarians prefer to perform desexing at 6 months of age, rather than including it in the initial health program of kittens, this puts the onus on owners to organize the procedure several months after the initial vaccinations. It is evident that many cat owners are not diligent at ensuring their cat is desexed at 6 months of age, as evidenced in a Western Australian survey where less than 50% of cats <2 years of age were desexed, and as evidenced by the number of unwanted kittens from owned queens surrendered to the RSPCA [6,7]. If veterinarians took responsibility for ensuring that kittens are desexed 2 to 3 weeks after their second vaccination (typically administered at 12 weeks of age), with suture removal at the time of the third vaccination (16 weeks of age), compliance would likely be greater, and the number of unwanted kittens being born to owned queens and subsequently euthanized would likely be reduced. In the current study, amongst those advocating the use of EAD, population control was the most common reason for using EAD (89% and 71% of respondents in 2015 and 2008, respectively), and this is supported in the scientific literature as a tool to control dog and cat populations [16,19].

### 4.1. Anesthetic Risk

Amongst respondents not advocating the use of EAD, a high proportion reported that anesthetic risk was a key reason for not using EAD. In a survey of Australian veterinarians, 75% considered that anesthetic safety was an inhibiting factor for EAD [32]. Safe anesthetic protocols for EAD have been described [34,35]. Four different anesthetic techniques were trialed with EAD (6–14 weeks of age) on 48 female and 48 male healthy kittens from animal shelters operated by the Massachusetts Society for the Prevention of Cruelty to Animals (SPCA) [35]. Anesthetic protocols were compared by evaluating pre-anesthetic disposition, depth of sedation, loss of resistance to handling, induction time, induction quality, analgesia and anesthesia without the use of an inhalational anesthetic (males) and extubation times (females), time to sternal recumbency, time to standing, and quality of recovery. All protocols were found to be safe with no deaths or serious anesthetic complications, and based on the study, the protocols recommended were 11 mg/kg of tiletamine/zolazepam intramuscularly (IM) for male kitten desexing, and midazolam 22 mg/kg IM and ketamine 11 mg/kg IM followed by intubation and inhalational isoflurane for female kitten desexing. Age appropriate protocols were used including withholding food only 4–8 h prior to surgery, and minimizing body heat loss by use of supplemental heat sources, minimizing hair clipping and alcohol preparation, use of warmed surgical scrub, minimizing visceral exposure, and reducing surgery times [35].

Similarly, ten different anesthetic regimens in dogs were evaluated during desexing of 49 male and 50 female pups at 6–14 weeks of age selected from animal shelters operated by Massachusetts SPCA [34]. As with the kittens, male pups were supplied inhalational isoflurane by mask if required, and females were intubated before being provided with inhalational anesthetic (isoflurane). It was found that in male pups, atropine 0.04 mg/kg and oxymorphone 0.22 mg/kg IM premedication and propofol 6.5 mg/kg IV induction was the best protocol. In female pups, atropine 0.04 mg/kg and oxymorphone 0.11 mg/kg premedication, and propofol induction 3.4 mg/kg IV was best. Again, no important anesthetic complications or deaths occurred with these protocols, indicating that puppies and kittens between 6–14 weeks can safely be anesthetized for surgical desexing procedures with the correct planning and management [34]. However, in pediatric humans, propofol was associated with a higher risk of death, and drug labelling indicates use only in children above 3 years of age [36]. In puppies (*n* = 25) and kittens (*n* = 34) less than 12 weeks of age, when alfaxalone was as an induction agent followed by intubation and maintenance with isoflurane (puppies and kittens) or as a sole agent (kittens), it resulted in acceptable induction, maintenance, and recovery, with measured cardiovascular and respiratory parameters well maintained [37,38].

In a case control study in the UK in 117 veterinary practices, anesthetic deaths in small animals were analyzed between 2002–2004 [39]. Increasing age of cats was associated with a higher risk of anesthetic mortality, as was poor health status and body weight above or below ideal. Importantly, cats in the lowest age category of 0–0.5 years had the lowest risk of death compared to all other age brackets. Cats <2 kg or >6 kg were at higher risk of anesthetic death. There was no increase in risk of death during or after EAD relative to other procedures, even though EAD patients would have been more likely to weigh less than 2 kg, but their low body weight would have been a result of age and not intercurrent disease (the latter increases the risk of anesthetic death).

### 4.2. Orthopedic Concerns

Some respondents selected increased risk of orthopedic conditions, particularly hip dysplasia, following EAD, but there is conflicting evidence in the literature regarding the association with EAD. In a retrospective study at the Society for Prevention of Cruelty to Animals New York [40], 1842 dogs underwent desexing between 6 weeks and 12 months. Dogs desexed when less than 5.5 months of age had a higher incidence of hip dysplasia and were diagnosed at an earlier age. However, a significantly lower percentage were euthanized for hip dysplasia compared with dogs desexed when older. This suggests that EAD may be associated with a less severe form of hip dysplasia. In contrast, when 635 dogs from two humane organizations were desexed <24 weeks (EAD) or ≥24 weeks of age (TAD) [41], of the 269 animals followed up, there was no difference in incidence of hip dysplasia between these groups. Musculoskeletal problems were observed in 8% of all animals, predominantly mild hip dysplasia not requiring surgical management. For dogs admitted as primary care and secondary and tertiary referral patients to the University of California veterinary teaching hospital, disease occurrence was compared between dogs left intact and those desexed in <6 months, 6–11 months, 1 year (12–23 months) and 2–8 years of age. Within both male and female Golden Retrievers, incidence of hip dysplasia was highest in those desexed when <6 months of age compared to those desexed when older or not desexed [23]. In contrast, within Labrador Retrievers, regardless of age at desexing, males had low incidences of hip dysplasia, and in females, incidences were similar within females desexed at various ages <2 years, but were significantly higher than in intact females. In another study from the same hospital, within German Shepherds, incidences of hip dysplasia were highest amongst those desexed when <12 months of age. In males, incidences were higher amongst those desexed when <6 months of age compared to those desexed when 6–11 months, while in females, incidences were similar in those desexed when <6 months of age and 6–11 months [22]. These results collectively suggest that desexing when <6 months of age rather than 6–11 months may increase the risk of hip dysplasia under some circumstances in some breeds of dogs. Other factors including body condition and body weight also influence the likelihood of dogs developing hip dysplasia and other skeletal problems [42,43] and desexing increases the risk of obesity [44]. However, none of these studies adjusted for body condition score after desexing and prior to diagnosis. Interpretation of results is further confounded because some of the patients were referred, and resolution of obesity is recommended in early management of hip dysplasia. It is possible that increases in the risk of hip dysplasia with EAD are due to increases in body condition, although one study reported reduced obesity in EAD dogs compared to TAD [40]. In contrast, another study of 1930 dogs found no long term difference in obesity in EAD compared to TAD dogs, and both had a greater risk of being overweight than intact dogs [45] Other factors affecting the hip joint, including hormonal factors, also may influence the risk of hip dysplasia [46]. Nevertheless, restricted feeding and lower body condition delays or prevents development of hip joint osteoarthritis in Labrador Retrievers, and is recommended following desexing for breeds at risk of hip dysplasia [47].

### 4.3. Other Orthopedic Problems

Cruciate ligament rupture was mentioned as a contributing reason that some respondents did not support EAD. Compared to intact animals, male and female Golden Retrievers had a higher incidence of cranial cruciate ligament rupture when desexed <6 months and between 6–11 months, and between 2–8 years in females [23,24]. Male Labradors desexed <6 months had a significantly higher incidence of cranial cruciate rupture and elbow dysplasia than intact animals [23]. This again indicates that desexing may have some adverse effects on canine joints, but no differences were found between desexing less than 6 months and 6–11 months. In male German Shepherds desexed <6 months and between 6–11 months, cranial cruciate ligament rupture occurred in 13% and 8%, respectively, compared to intact males (<1%). Similarly in female German Shepherds desexed <6 months of age, cranial cruciate ligament rupture occurred in 5% females and in 8% of those desexed 6–11 months compared to <1% of intact females [22].

In a controlled trial, 31 healthy kittens were randomly assigned desexing procedures at either 7 weeks or 7 months old, or were left as intact controls [12]. There was no significant difference between those desexed at 7 weeks or 7 months in physeal closure time, but at both desexing ages, closure times were delayed compared to the intact controls. There was no significant effect of age of desexing on incidence of fractures [12]. In a larger study of 783 cats, there was no correlation between delayed physeal closure and fractures in cats [48]. Male desexed cats (359) had delayed closure of the greater trochanteric, distal femoral, and tibial tuberosity physes compared to entire males (95) based on pelvic and femoral radiographs, but there was no significant difference in female cats (237 desexed, 92 entire). The effect of EAD was not assessed separately [48]. 

Collectively, these results suggest that in dogs, but not in cats, desexing may contribute to an increased risk of some orthopedic problems such as hip dysplasia and cranial cruciate ligament rupture, and there is some evidence that for some breeds, dogs desexed under 6 months may have increased risk compared to those desexed between 6 and 11 months or later. Further prospective studies are required to confirm these findings.

### 4.4. Neoplasia

Although none of the respondents in the current study mentioned increased risk of neoplasia as a concern, it is often raised in forums discussing EAD. While a number of studies have found incidences of some neoplasias are increased in desexed dogs compared to entire animals, or animals desexed after a year of age, no studies were found that reported increased risk of non-mammary neoplasias for EAD compared to TAD. For example, female Golden Retrievers that were desexed at any age had significantly higher incidence of one or more types of cancer compared to intact females. Lymphosarcoma was significantly increased in those desexed ≤6–11 months compared to intact animals, and those desexed between 2–8 years had an increased incidence of mast cell tumors [23]. Conversely, Golden Retriever females desexed ≥12 months had a higher incidence of hemangiosarcoma compared to those desexed <12 months and intact animals, whereas Golden Retriever males desexed <12 months had a higher incidence of lymphosarcoma [24]. A companion study in German Shepherds did not find an increase in risk of lymphosarcoma, hemangiosarcoma or osteosarcoma in dogs desexed at <6 months or 6 to 11 months, compared to those desexed at older ages, and to intact animals [22]. 

### 4.5. Mammary Tumors

A minority of our respondents cited mammary tumors as a reason for supporting EAD. It is well documented that desexing before the first estrus significantly reduces the risk of mammary carcinomas in dogs and cats. In a retrospective case-control study, involving 71 dogs with adenocarcinoma, 22 dogs with malignant mixed-mammary tumors and 87 matched controls, desexed bitches were reported as having only 12% of the risk of mammary cancer compared to intact bitches [49]. Bitches desexed before their first estrus were reported as having 0.5% of the mammary cancer risk, animals that had one estrus cycle were reported as having 8% of the risk and those that had two or more estrus cycles were reported as having 26% compared to intact bitches. In a case-control study of 147 dogs diagnosed with mammary carcinoma or adenocarcinoma and 131 non-cancer control dogs, desexing <1 year of age was strongly protective against mammary cancer, reducing odds by >90% compared to intact females, but effects of EAD versus TAD were not assessed [44].Thin (rather than not thin) body condition at 9–12 months reduced the risk of mammary cancers among desexed animals by 96%, but not significantly so among intact animals (odds ratio 0.6), highlighting the complex interaction with other risk factors such as obesity. However, the benefits of EAD in reducing risk of mammary tumors would be less important if the incidence of mammary tumors is low. In a recent study in German Shepherds [22], percentages of dogs diagnosed with mammary carcinomas varied between the desexing age groups from 0 to 5%, and only occurred in females desexed at 6 months or older and intact females, but incidences could not be calculated as follow-up periods were not reported. 

### 4.6. Urinary Incontinence

A minority of our respondents indicated that urinary incontinence in dogs was a contributing reason to why they did not advocate EAD. Results of studies are conflicting on the influence of EAD on the occurrence of urinary incontinence in female dogs. In a 5 year study of bitches, questionnaire data was provided by 16 of 233 randomly selected practicing veterinary surgeons in the UK. Urinary incontinence occurred in 9 (10%) of the 92 bitches desexed before the first estrus, 8 (3.7%) of the 218 bitches desexed after first estrus, and in 4 (1.5%) of the 259 intact bitches [50]. However, the 7% response rate to the survey could have biased the results. A study compared 202 desexed bitches diagnosed with urinary incontinence with 102 randomly selected control dogs from the target population [51]. Age of the dog at time of desexing did not significantly affect the development of urinary incontinence, based on owner questionnaires determining age at desexing, clinical signs and diagnosis. In contrast, another study found decreasing age of desexing was associated with an increased risk of urinary incontinence in female dogs [40]. Dogs desexed <3 months of age were at higher risk than those desexed ≥3 months. A recent study in 1170 German Shepherds found no occurrence of urinary incontinence in intact females, but the percentage affected in females desexed at <6 months was 4.7% and for those desexed at 6–11 months the percentage affected was 7.3%, which was a significant increase compared with intact females [22] 

### 4.7. Urinary Obstruction in Male Cats

None of our respondents mentioned urinary obstruction in male cats as a reason for not advocating EAD, and concerns that EAD predisposes male cats to lower urinary tract disease—in particular, urolithiasis—are not supported by results from multiple studies [5,29]. In a study of 263 cats from animal shelters that were randomly assigned to be desexed <24 weeks or ≥24 weeks, cats desexed <24 weeks did not develop any signs of urethral obstruction, whereas 2 male cats desexed at the traditional age did, based on a telephone survey 30–60 months after surgery [5]. 

In a retrospective study of causes of lower urinary tract disease, no association was found between age of castration and development of urolithiasis in 112 male cats castrated before or after 6 months of age. Incidence increased with increasing cat age, and hence the incidence was higher in castrated animals compared to entire cats due to their older average age [29]. Animals diagnosed with urolithiasis, urethral calculi, urethral obstruction, urethritis, urethral stricture, cystic calculi or cystitis were included. Factors that were associated with the development of disease included obesity and cat age.

### 4.8. Ease of Surgery

Of those teaching staff in 2015 who advocated EAD, 78% stated ease of surgery (including better visualization, more elastic tissue, and less bleeding) as a major reason. Similar surgical benefits of EAD in cats and dogs were identified when 775 cats and 1213 dogs were randomly assigned an age for desexing (<12 weeks, 12–23 weeks and >23 weeks) and complications during anesthesia, surgery and the immediate post-operative period (7 days post-surgery) recorded. Pre-pubertal desexing did not increase risk of adverse outcomes or mortality when appropriate protocols are used. Furthermore, animals desexed >23 weeks of age had significantly more minor adverse outcomes associated with surgery, such as poor healing of the surgical incision. Animals <12 weeks of age had a significantly shorter surgical time [28]. This was consistent with the reason some teaching staff in the current study advocated EAD. A retrospective study assessed post-surgical complications in a shelter desexing program involving 312 healthy female cats weighing 1 kg or more [52]. Surgeries were performed at four veterinary clinics. Wound complications post-surgery were not age associated, and there was no significant difference between groups desexed ≤12 weeks or >12 weeks [52].

### 4.9. Behavioural Effects

In the current study, positive behavioral changes were mentioned as a reason for advocating EAD by 33% of staff in 2015. In a retrospective cohort study of animals adopted from a New York SPCA shelter, behavior in dogs desexed <5.5 and 5.5–12 months of age were assessed 3 months to 11 years after adoption. Prevalence of noise phobias and sexual behaviors increased with a decreasing age of desexing, while prevalence of escaping behaviors, separation anxiety and urination in the house decreased [40]. Male dogs desexed <5.5 months also had a higher prevalence of aggression towards family members than those desexed 5.5–12 months of age, but no significant association with serious aggression was detected. A common reason dogs are surrendered to animal shelters is for behavioral problems, including escaping behaviors, inappropriate elimination and aggression, and the first two were decreased with EAD [40]. In a related study from the same shelter, the long-term effects of EAD were evaluated in 1660 cats desexed at <1 year [53]. Cats desexed <5.5 months of age had lower incidences of aggression towards veterinarians, sexual behaviors and urine spraying, but an increase in hiding behaviors. However, confidence in the findings from both of these studies are limited because animals were not randomly selected as puppies and kittens, and then allocated to EAD or traditional age or older. Puppies and kittens are more likely to be adopted than animals older than 6 months, and therefore, minor behavioral issues such as timidness are more likely to be acceptable in a puppy than an older animal, where there is less demand. Secondly, many of the undesirable behaviors that occur in adults such as phobias, sexual behaviors, aggression or timidness are not evident or problematic in puppies and kittens, and therefore it is easier to select against these behaviors in older animals. This is particularly so in older cats, where the euthanasia rate for adult cats in shelters can exceed 80–90%, and only adult cats with exceptionally good temperament get adopted.

In another study which addressed this limitation, behavior of EAD kittens was evaluated when 800 healthy kittens in Flanders, Belgium, were randomly assigned to EAD or traditional age desexing [16]. Surveys were completed by adopters at 2, 6, 12, 18 and 24 months after adoption, and there was no difference between EAD and traditional age desexing with regards to undesirable behaviors such as inappropriate elimination, fearful behavior, aggression and destruction.

### 4.10. Teaching EAD

In our study, very few veterinary teaching staff advocated EAD when teaching students, and a minority of veterinary students were provided with opportunities to witness or perform EAD procedures before graduation. In a recent study of Australian veterinarians, those who were younger and graduated more recently were more likely to perform desexing at the traditional age of 6 months than earlier, and there was a significant influence of university of graduation on their view of desexing age [32]. In our study, median time since graduation for teaching staff was 25 years and most were in the mid-range of seniority (lecturer and senior lecturer), and this coupled with the relatively low numbers of staff teaching desexing across Australian and New Zealand universities, prevented assessment of the effect of time since graduation and university of graduation, on attitudes to EAD. 

In a study of 4th year veterinary students at Texas A&M university, desexing teaching programs that included EAD provided students with improved surgical skills, increased understanding of functions and goals of humane organizations, awareness of pet overpopulation problems and what students as future veterinarians can do about it [54]. Therefore, it would be beneficial for Australian and New Zealand veterinary students to have the opportunity to learn about these benefits associated with EAD, and develop the skills required to perform the procedure. However, additional training would be necessary for students to graduate with the skills to perform EAD, as they would require sufficient competency to perform the desexing procedure quickly. Given the budget deficits within veterinary schools in Australia, the cost of surgical training of students, and limited access to suitable caseloads, welfare agencies could partner with veterinary schools to facilitate students gaining this experience. The type of exposure veterinary students gain in their training in regards to EAD likely also depends on school policy relating to the age desexing is recommended to clients in the university teaching clinic. If schools reviewed this policy, and recommended to owners of cats that desexing be performed as part of their initial health program, this would also assist with increasing students’ exposure to EAD. 

### 4.11. Limitations of Study

The precision of results and the external validity of this study were limited by the number of respondents. The sample size was small, but was representative of the population under study i.e., Australian and New Zealand staff directly teaching anesthetic and surgical aspects of desexing to students, because 89% (25/28; 2015) and 79% (15/19; 2008) of staff identified as teaching desexing, and subsequently confirmed by questionnaire responses, completed the survey. Because we aimed to compare responses between 2008 and 2015, questionnaire questions and age categories remained the same except for additional questions in 2015. It was not determined what other exposure to EAD students gained through other units of their studies and extra-curricular activities not directly involved with teaching desexing, because this was not addressed by the questionnaire. Students may be learning different aspects of EAD in other practical classes, professionalism courses, welfare subjects and volunteer work. Furthermore, school policies in their teaching hospitals were not evaluated in this study, which may also impact what staff are teaching to students. 

## 5. Conclusions

Our study found that the majority of relevant veterinary teaching staff in Australia and New Zealand are not advocating EAD to students regardless of species or sex of animal, and the number advocating EAD decreased from 2008 to 2015. Teaching staff feel that shelter animals are more suitable than client-owned animals for the procedure, which is not logical with regards to concerns about subsequent adverse health effects, given that shelter animals are subsequently adopted by clients. Concerns about increased anesthetic risks and higher incidences of orthopedic conditions associated with EAD in cats is unsupported in current literature. This failure to advocate EAD for cats in teaching of veterinary students may be contributing to the large number of kittens from owned queens being surrendered to shelters and euthanized. Differences between views of some participants and published evidence about adverse effects of EAD was surprising, especially for cats and for anesthetic complications, because it is expected that, as educators, veterinary teaching staff are well informed of the scientific literature. Only a minority of veterinary students in Australia and New Zealand are graduating with hands-on experience to facilitate them using it after graduation, Therefore, it is recommended that, to increase the number of veterinarians graduating with hands-on skills of EAD, animal welfare agencies partner with veterinary schools to provide this training, and that school policy on age of desexing in their teaching hospital be reviewed to include recommending desexing of cats towards the end of the initial vaccination program. Given that only a minority of teaching staff had current experience with desexing puppies or kittens 4 months of age or younger, it is recommended that veterinary teaching staff gain competence in EAD. Further well-designed, prospective studies in dogs comparing EAD and TAD are required to have more confidence about the increased risk of some of the reported adverse effects. 

## Figures and Tables

**Table 1:** Responses (number and percentage) of university veterinary teaching staff from Australia and New Zealand in each of 2008 (*n* = 15) and 2015 (*n* = 25) to the question ‘For all factors considered, including safety and population control, what age do you think is best to desex each of the following *categories*’ Categories were client-owned and shelter female and male cats and dogs.

		≤3 Months	4–5 Months	≥6 Months	% ≤5 Months	Difference (2015 Minus 2008)	95% Confidence Interval for Difference	*p* for Difference ^1^
Female Cats								
Client-owned	2015	4% (1)	64% (16)	32% (8)	68%	−25%	−52% to 0%	0.087
	2008	7% (1)	87% (13)	7% (1)	93%			
Shelter cats	2015	44% (11)	32% (8)	24% (6)	76%	−24%	−44% to −4%	0.044
	2008	60% (9)	40% (6)	0% (0)	100%			
Male Cats								
Client-owned	2015	0% (0)	68% (17)	32% (8)	68%	−19%	−48% to 16%	0.202
	2008	7% (1)	80% (12)	13% (2)	87%			
Shelter cats	2015	44% (11)	32% (8)	24% (6)	76%	−24%	−44% to −4%	0.044
	2008	67% (10)	33% (5)	0% (0)	100%			
Female Dogs								
Client-owned	2015 ^2^	0% (0)	30% (7)	70% (16)	30%	−23%	−68% to 18%	0.139
	2008	0% (0)	53% (8)	47% (7)	53%			
Shelter dogs	2015	32% (8)	32% (8)	36% (9)	64%	−23%	−54% to 12%	0.112
	2008	40% (6)	47% (7)	13% (2)	87%			
Male Dogs								
Client-owned	2015	0% (0)	32% (8)	68% (17)	32%	−8%	−46% to 30%	0.619
	2008	7% (1)	33% (5)	60% (9)	40%			
Shelter dogs	2015	44% (11)	32% (8)	24% (6)	76%	−24%	−44% to −4%	0.044
	2008	53% (8)	47% (7)	0% (0)	100%			

^1^ These *p*-values are to assess the null hypotheses that there was no difference in the proportions between 2008 and 2015 for the respective subset of animals for the question ‘For all factors considered, including safety and population control, what age do you think is best to desex each of the following categories’. They are exact two-tailed mid-p *p*-values disregarding correlations in responses for the 4 subjects who responded in both years; ^2^ N = 23 respondents; the other two respondents stated that the best age depends on the dog’s breed.

**Table 2:** Responses of university veterinary teaching staff from Australia and New Zealand in 2008 and 2015 to the question ‘In your personal opinion of early-age desex, do you advocate its use in…’.

Year Questionnaire Completed	No. Respondents	% (No.) Responding ‘Yes’	Difference (2015 Minus 2008)	95% Confidence Interval for Difference	*p* for Difference ^1^
Female Cats					
2015	25	32% (8)	−28%	−64% to 1%	0.077
2008	15	60% (9)			
Male cats					
2015	25	32% (8)	−28%	−60% to 9%	0.077
2008	15	60% (9)			
Female Dogs					
2015	25	20% (5)	0%	−30% to 29%	0.843
2008	15	20% (3)			
Male dogs					
2015	25	20% (5)	−27%	−64% to 12%	0.060
2008	15	47% (7)			

^1^ These *p*-values are to assess the null hypotheses that there was no difference in the proportions between 2008 and 2015 for the respective subset of animals. Staff who completed the questionnaire in both years were excluded from these comparisons.

**Table 3:** Responses of university veterinary teaching staff from Australia and New Zealand in 2008 and 2015 to the question ‘In your teaching of desexing to students of both female and male cats and dogs, are you advocating the use of early age desexing (EAD)?’.

Year	No. Respondents	% (No.) Responding ‘Yes’ ^1^	% (No.) Responding ‘No’ ^1^	% (No.) Responding ‘Yes’ and ‘No’ ^1^
2015	25	24% (6)	64% (16)	12% (3)
2008	15	20% (3)	53% (8)	27% (4)

^1^ ‘Yes’ meaning staff do advocate the use of EAD in their teaching and ‘no’ indicating they do not advocate EAD in teachings to students.

**Table 4:** Reasons university veterinary teaching staff from Australia and New Zealand in 2008 and 2015 advocated early age desexing; the percentages (and numbers) of respondents nominating each response within each year are shown, expressed as percentages of those answering ‘yes’ or ‘yes and no’ to the question ‘In your teaching of desexing to students of both female and male cats and dogs, are you advocating the use of EAD?’. Each respondent could nominate multiple reasons.

Reason for Advocating EAD	2015 (*n* = 9)	2008 (*n* = 7)
Population control	89% (8)	71% (5)
Ease of surgery-better visualisation, more elastic tissue, less bleeding	78% (7)	29%(2)
Positive behavioural changes	33% (3)	29% (2)
Quicker recovery	44% (4)	14% (1)
Decreased risk of one or more medical problems—obesity, mammary tumors	11% (1)	14% (1)
No reason(s) given	0% (0)	14% (1)

**Table 5:** Reasons university veterinary teaching staff from Australia and New Zealand in 2008 and 2015 did not advocate early age desexing: the percentages (and numbers) of respondents nominating each response within each year are shown, expressed as percentages of those answering ‘no’ or ‘yes and no’ to the question ‘In your teaching of desexing to students of both female and male cats and dogs, are you advocating the use of EAD?’. Each respondent could nominate multiple reasons.

Reason for Not Advocating EAD	2015 (*n* = 19)	2008 (*n* = 12)
Anesthetic risks (including more difficult to perform)	63% (12)	92% (11)
Increased risk of one or more medical problems including hip dysplasia in dogs and urinary incontinence in female dogs	47% (9)	54% (6)
Hypoglycemia	32% (6)	58% (7)
Difficulty of surgery	5% (1)	33% (4)
Hypothermia	5% (1)	17% (2)
School policy	10% (2)	8% (1)
No reason(s) given	0% (0)	8% (1)

**Table 6:** Distribution of eight Australian veterinary schools by opportunities for their veterinary students to see and or perform EAD before graduation in 2015 ^1^.

Student Exposure to EAD	Proportion of Universities Where Students Witness EAD Procedures	Proportion of Universities Where Students Perform EAD Procedures
Majority of graduating students	37% (3)	25% (2)
Minority of graduating students-depends on availability of suitable animals	13% (1)	25% (2)
No exposure before graduation	50% (4)	50% (4)

^1^ These categories are based on the proportions of students who have had the chance to perform or witness EAD while on internal rotations or practical classes. They are not based on whether students had additional opportunity when on external rotations or volunteer placements.

## Supplementary Materials

The following are available online at www.mdpi.com/2076-2615/8/1/3/s1, Table S1: Attitudes of veterinary staff teaching toward early-age gonadectomy (EAG): Questionnaire questions.

## Abbreviations

The following abbreviations are used in this manuscript:

EAD	Early age desexing
TAD	Traditional age desexing
