# Supplementary material for: Attitudes of Veterinary Teaching Staff and Exposure of Veterinary Students to Early-Age Desexing, with Review of Current Early-Age Desexing Literature
Source: Animals (Basel). 2017 Dec 25;8(1):3. doi: 10.3390/ani8010003 (PMC5789298; doi:10.3390/ani8010003)
Supplement: Supplementary file 1 [file animals-08-00003-s001.pdf]

Table S1. Attitudes of veterinary staff teaching toward early-age gonadectomy (EAG): Questionnaire questions.

1. In what year did you graduate from your veterinary degree?
2. What university did you graduate from?
3. What is your teaching position within the vet school?
4. How many hours do you teach? (lecturing/practical classes)
5. How many hours do you perform surgery, if any? (i.e., client owned animals)
6. How many contact teaching hours do you have on the topic of desexing?
7. In your teaching of desexing, does it include:

   ☐ Surgical theory
   ☐ Anesthetic theory
   ☐ Other formal theory
   ☐ Practical teaching

8. To what years of veterinary students do you teach the theory/practical aspects of desexing?

   ☐ 1st year
   ☐ 2nd year
   ☐ 3rd year
   ☐ 4th year
   ☐ 5th year

9. For all factors considered, including safety and population control, what age do **you think is best** to neuter for each of the following categories:

   (Please identify one age group for each line)

   Client owned:

   Female cats: ≤3 months, OR 4–5 months, OR ≥6 months
   Male cats: ≤3 months, OR 4–5 months, OR ≥6 months
   Female dogs: ≤3 months, OR 4–5 months, OR ≥6 months
   Male dogs: ≤3 months, OR 4–5 months, OR ≥6 months

   Animals being re-homed for shelter:

   Female cats: ≤3 months, OR 4–5 months, OR ≥6 months
   Male cats: ≤3 months, OR 4–5 months, OR ≥6 months
   Female dogs: ≤3 months, OR 4–5 months, OR ≥6 months
   Male dogs: ≤3 months, OR 4–5 months, OR ≥6 months

10. In your **personal opinion** of early-age desexing/EAG, do you advocate its use in:

   Female cats—YES/NO
   Male cats—YES/NO
   Female dogs—YES/NO
   Male dogs—YES/NO

   If answered no, under what situations would you support EAG?

11. In your **teaching** of desexing of both female and male cats and dogs, are you advocating the use of EAG?

   YES/NO

   If yes, why?

   ☐ School policy/support

- ☐ Population control
- ☐ Benefits
    - ☐ Less bleeding
    - ☐ Behavioral changes
    - ☐ Decreased asthma
    - ☐ Decreased gingivitis
    - ☐ Decreased urine spraying
    - ☐ Decreased obesity
- ☐ Better visualisation
- ☐ More elastic tissue
- ☐ Less stitching
- ☐ Fewer drugs required
- ☐ Quicker recovery
- ☐ Other—please elaborate.

If no, why?

- ☐ School policy/beliefs
- ☐ Risks
    - ☐ Anesthetic
    - ☐ Hypoglycaemia
    - ☐ Bleeding
    - ☐ Seroma
    - ☐ Hip dysplasia
    - ☐ Inappropriate elimination
    - ☐ Cystitis (dogs)
    - ☐ Urinary incontinence (dogs)
    - ☐ Urinary tract obstruction (cats)
- ☐ Difficulty
    - ☐ Hands too big
    - ☐ Unfamiliarity
    - ☐ Convincing owners
    - ☐ Friability of tissue
- ☐ Other—please elaborate.

12. About how many kittens, 4months or younger, have you desexed in the last 12 months **without** students?
13. About how many kittens, 4 months or younger, have you desexed in the last 12 months **with** students?
14. About how many puppies, 4months or younger, have you desexed in the last 12 months **without** students?
15. About how many puppies, 4 months or younger, have you desexed in the last 12 months **with** students?
16. In your opinion, do you believe that implementing routine desexing of client-owned kittens before 4 months of age would result in a measurable reduction in the number of unwanted kittens from owned queens that are surrendered to shelters?

    YES/NO

    Please give reasons to support your answer
17. In your experience, what is the approximate proportion of owned cats that have at least one litter of kittens before they are desexed?

    - ☐ >20–40%
    - ☐ >40–50%
    - ☐ >60–80%
    - ☐ >80–100%

18. In your experience, what is the approximate proportion of unwanted kittens from owned queens compared to stray queens?

   ☐ >20–40%
   ☐ >40–50%
   ☐ >60–80%
   ☐ >80–100%

19. What proportion of students have the opportunity to observe an early age desexing procedure at the university you teach at?
20. What proportion of students have the opportunity to perform early age desexing at the university you teach at?
